# Tutorial Action and Emotional Development of Students as Elements of Improved Development and Preventing Problems Related with Coexistence and Social Aspects

**DOI:** 10.3390/ejihpe10020045

**Published:** 2020-05-23

**Authors:** Jorge Expósito López, Ramón Chacón-Cuberos, María Elena Parra-González, Eva María Aguaded-Ramírez, Alfonso Conde Lacárcel

**Affiliations:** Department of Research Methods and Diagnosis in Education, University of Granada, 18071 Granada, Spain; jorgeel@ugr.es (J.E.L.); rchacon@ugr.es (R.C.-C.); eaguaded@ugr.es (E.M.A.-R.); alfconl8@ugr.es (A.C.L.)

**Keywords:** education, emotions, teaching role, tutoring

## Abstract

Integral development of students is promoted through tutorial action. Tutorial action is understood as the personal development of students, their learning, and their capabilities for social and labour integration. A descriptive, nonexperimental and ex post facto design was used. The sample consisted of 569 primary school students. The importance of emotional education and student tutoring was highlighted by the results produced. A relationship was established between working on emotions and emotional regulation, cognitive re-evaluation, and capacity to respond in an emotionally appropriate way when faced with different situations.

## 1. Introduction

Current society is constantly changing at all levels. These changes must be reflected in education provision, as education must go hand in hand with other societal themes in order for a global approach to be taken. These change processes must be considered through collaborative and shared experiences which enable student experiences and actions to be transformed [1].

A response to this challenge comes in the form of establishing procedures for analysis and improvement as an unavoidable responsibility of contemporary university teaching staff. These procedures will enable teaching-learning processes to meet the challenges posed to universities by the university and social context of the 21st century. Nevertheless, these situations must be considered through careful reflection, planning, and verification of the suitability of proposed changes, since this is the only way in which students, teachers, and the institution itself can be expected to adapt to them. Intervention must go beyond mere limited and punctual actions, offering a whole experience of change [2,3].

Both educational systems and students undertaking their studies will be affected by these changes. At the same time, these same two agents and their associated needs also provoke these changes. Students need training processes to be adapted and improved, with guidance, tutoring, and tutorial action playing a central role. Further, a new approach to this aspect must be considered when educating and guiding students. This should be based on tutorial action and incorporate much broader global content. The tutorial action of teachers covers all actions, which make up educational actions. It runs in a parallel and complimentary way to the development of the educational curriculum [1,2,3]. Further, the relevance of student guidance and tutoring before, during, and after the educational process has been revealed as one of the fundamental elements of this training process. This is true in the sense of both the contents they develop and the outcomes they permit individuals to achieve [4].

Taking a more traditional perspective, tutoring actions performed during tutoring must be transcendental, whilst also including a time-limited commitment to resolving small coexistence problems associated with school life, such as minor conflicts, physical and verbal victimization, or deterioration of facilities and material, as well as communicating the results of evaluative tests to families. Further, tutoring action should be considered as a central time commitment and key aspect of teaching work. This will facilitate the personal development of students, their learning, and their capabilities for social and labour integration [3,5].

In other words, tutorial action must be considered as an appropriate element or setting for improving the educational management of content. This content is repeatedly referred to as transversal content. Nevertheless, it plays a fundamental role in the development of educational processes and lifelong training of the students involved in these processes [6].

Pedagogical theories that focus their study on global educational processes and lifelong education reveal the importance of student emotions and affect in these processes, in that they contribute to the improved academic and social education of students [7]. In other words, a better regulation and management of emotions and affect provoke integral improvements to personality development at an individual level, and to society overall [8,9]. As a result, they enable fundamental outcomes to be achieved in relation to training processes, and social and labour integration as an active member of society.

In addition, the approach of the term tutorial action is considered to be an educational element which contributes to the integral development of students [10]. This is related with improvements in different areas of intervention: teaching how to learn to get to know each other, teaching how to learn to coexist, teaching how to learn how to learn, and teaching how to learn to make decisions. Thus, we can say that tutorial action represents one of the best routes through which emotional education can be addressed in schools [3]. In this way, it also becomes an educational innovation in itself, responding to the social needs that are not met through ordinary academic materials. From this new conceptualization, tutorial action is not only relevant to the relationship of teaching staff with students and their families, and its resultant effect on students’ academic performance, but it also forms a central educational element which permits integral training processes to be articulated, thus fostering the personal and social development of students and their emotional education in this process.

In this sense, two models of tutorial attention can be differentiated. The first of these is more traditional in nature and tends to dedicate only limited and specific time to tutorial action. This occurs via specific timetabled classes, which dedicate attention to relevant themes in a parallel way to the curriculum, as opposed to integrating themes within the curriculum. Classes refer to transversal content, with students being mere spectators and students’ families being occasional spectators. Within this type of model, tutorial action is a complementary action, which exercises little influence over training processes [2,3].

Nevertheless, current models of tutorial action correspond to action at any time and place. They consider the educational process as a constant that takes place in the classroom and beyond, dealing with themes of personal development, social learning, and integration. Rather than taking place parallel to the curriculum, these themes are integrated into the development of each and every one of the curricular disciplines. In this way, tutorial action within this model provides the foundation from which transversality can be increased and a setting within which all of these aspects can be developed. This is sometimes not considered, despite being so important for education at a basic level and in all of its disciplines. Within the educational processes employed by these models, the entire educational community becomes an active agent, facilitating social integration and social skill development, and increasing the possibility of establishing much more effective integral educational processes [1,2,6].

The present research work leans on these two models, using them to provide a comparative analysis between groups. It examines the emotional development of students according to the two models of tutoring. Equally, it strives to consider the emotional aspects that must be borne in mind, focusing interest on the emotional regulation of students.

Key aspects covered by Gardner’s theory of multiple intelligences and Goleman’s theory of emotional intelligence should be worked on as training content via tutorial action. Content should also be taken from theories which specifically advocate working in these ambits through guidance, tutoring, and the close attention afforded through tutorial action. As a result, both educational and work settings are interconnected. Child personal world should also be added to this consideration given that self-esteem is responsible for many academic successes and failures [11,12].

In other words, emotions and education go hand in hand, as good emotional education is necessary for all children. On the other hand, training and educational processes cannot be approached without considering the emotional aspects of the individuals integrated in the process. This includes both students as users, alongside teachers as managers of the aforementioned processes [6,12].

Thus, neither education nor training depend on traditionally considered factors such as intelligence. Instead, educational success is much more dependent upon the emotional state of individuals and the emotions with which they experience training processes. The foundation of this consideration is based on the fact that having a high intellectual quotient, is not enough to guarantee success in life. Indeed, something more than abstract intelligence is required in order to solve the personal problems generated by emotional responses and interpersonal relationships [13]. For this, development of a series of emotional intelligence skills is necessary. This will not be achieved through academic, abstract, or intellectual tasks, but by learning to control emotions and knowledge of the way the people we live with express themselves [14].

In this sense, education must consider emotions; however, this consideration must not take place in a general way. Alternatively, it should attend to each individual component and the way in which individuals manage them; this can be denominated emotional intelligence. Emotional intelligence involves a series of interrelated components [15]:

(a) Emotional perception and expression or conscious recognition of our emotions, knowing what we feel and how to verbalise this;

(b) Emotional facilitation or being capable of generating feelings that are favourable to thought;

(c) Emotional understanding around integrating these feelings within thought and being aware of the complexity of emotional changes; and 

(d) Emotional regulation or controlling emotions, both positive and negative, directing them and managing them effectively.

Of the aforementioned components, the present article deals with emotional regulation. This is with good reason given that emotions and the regulation of emotions are of great importance to the development and efficiency of learning processes. Thus, in one of its earliest definitions, emotional intelligence is linked to goals that can achieve "a series of intrinsic and extrinsic processes responsible for monitoring, evaluating and modifying emotional reactions, especially with regards to their temporal elements and intensity for reaching personal objectives" [16]. As has been stated by Pena et al. [17] and Gross at al. [18], the progression of research on emotional regulation has led to the definition of the term being revised and enrichened. Nevertheless, early established recommendations have been maintained and respected. Thus, emotional regulation is the process through which individuals identify, modulate, and express their emotions, whilst they interact with individuals or situations with the aim of reaching goals and objectives, adapting to a context or achieving personal and social well-being [19,20,21].

Interest in emotional regulation has increased in recent years as it is linked to well-being, health, personal development, performance, empathy, and interpersonal relationships [22,23]. Definitively, the handling or management of emotions themselves is fundamental for emotional well-being and healthy human functioning [21].

The present study analysed two fundamental aspects of emotional regulation, namely, expressive suppression and cognitive re-evaluation. The former characterises individuals who employ suppression or expressive inhibition strategies in order to modulate their emotional responses, inhibiting their behavioural expression [24] and suppressing their internal emotions when faced with unfavourable situations which generate discomfort [25]. The latter aspect is seen in individuals who use cognitive re-evaluation strategies to tackle stressful situations with a positive attitude, reconsidering what is considered as stressful and striving to quash discomfort.

Specifically, during childhood and adolescence, appropriate emotional management enables individuals to inhibit inappropriate impulses, redirect behaviour in a constructive way, and adapt to situations. This aspect is also key to the success of interpersonal relationships, overcoming complex situations, goal achievement and, consequently, achieving positive psychological and emotional well-being [26].

In a more specific way, children aged between 5 and 9 years old who work on emotional and social intelligence at educational centres improve their prosocial orientation. This is defined by aspects such as generosity, empathy, and collaboration. At the same time, these children reduce their aggressive behaviours and improve their mood in relation to concepts such as happiness and optimism. From the age of 12 onwards, these children experience less withdrawal, increased emotional self-knowledge, and greater capacity to handle stress.

In this sense, the tutorial action can constitute an essential way for the personal and academic development of the students. Based on this, there are various elements that must be considered according to Expósito [5], as shown in Table 1:

Based on all the above, as well as the reviewed scientific literature, the following research question could be established: Do those students who experience a holistic tutorial action have a better capacity for emotional regulation? In fact, the relationship between the type of guidance and tutoring delivered by teaching staff and the emotional development of their students is a relevant research topic in education, due to the aspects it may influence.

The present study, therefore, poses the following objectives:

- To know the perception of the students about the activities worked from the tutoring in the school.
- To examine the association between the tutorial action delivered by teaching staff and the emotional development of their students.

Further, it will specify this association in order to explain the relationships produced between the development of tutorial action in the educational context, its different indicators, and emotional development. Thus, the following research hypothesis can be established: Those students who work on the different components of the current tutorial action in class will have a better capacity for emotional regulation.

## 2. Design and Method

In order to carry out the present study, a descriptive, nonexperimental and ex post facto design was implemented in order to analyse outcomes once the phenomenon had occurred. Measurements were taken from within only a single group at a single time point, with the main intended outcome being to describe a concrete reality [27,28]. This was appropriate given that the purpose of the study is linked to the improvement of a specific context, whilst also recognising its use as an example of change in similar contexts.

### 2.1. Population, Sampling, and Participants

The reference population of the present study relates to students aged between 8 and 13 years old, undertaking compulsory primary education at public centres in the province of Granada and the autonomous cities of Ceuta and Melilla (Spain).

Sampling recruited a sample of volunteers and was therefore nonrandom in nature. It was performed in two stages. In the first stage, relevant settings were approached by presenting the project to educational centres’ School Councils. The research group approached centres with whom they had a prior relationship in order to verify their interest and secure their permission. In the second stage, natural groups or class groups were approached who had also expressed interest in participating in the study. Relatives of the students authorised this approach. A conditioned preselection was conducted of second- or third-year primary school groups. It was deemed that students at this age are mature enough to complete instruments and that they were authorised to participate in the study by their relatives and guardians. In the third and final phase, selected groups were explained the purpose of the research, and the procedure and instruments used for data collection in order to finally proceed to their administration.

The study obtained a valid sample of n = 569 schoolchildren coming from eight public centres, with self-reported ages of between 8 and 13 years old (mean = 10.39; SD = 0.95). The sample had a normalised gender distribution for this type of populational extract. Specifically, this amounted to 298 (52.3%) males and 271 (47.7%) females, providing a degree of verification regarding representativeness of the sample.

### 2.2. Data Collection Instruments

Suitability of instrument use with the type of students selected for the study sample was previously verified using a limited group which did not participate in the final study. This served to confirm full understanding of items and the response scale. The following data collection instruments were employed:

1. Self-registration sheet, collects demographic data such as gender, age or school year, educational centre, and characteristic information relating to tutoring, such as weekly dedication of hours to tutoring and the type of activities engaged in. These data are common in registers and are collected through broader data collection instruments for aspects related to the guidance and tutoring of students [3]. The measurement of the actions associated with the tutorial action considered seven items of a dichotomous type [(1) = Yes; (2) = No]. These items are: Dedication; Emotions; Group Work; Socialization; Learning and Study; Coexistence; Satisfaction), which were answered by the students. Participants had to respond considering affirmatively or negatively that the stated premise was fulfilled. The “Dedication” item considered whether schoolchildren spent at least 1 hour a week working on tutoring, while the “Satisfaction” variable considered whether the students were satisfied with the content worked on. The rest of the variables made reference to whether the students worked on the contents mentioned in each item-variable. The question used for each item is attached below:
  - Dedication: Do you dedicate at least 1 hour a week to tutoring?
  - Emotions: Do you do activities about your emotions?
  - Do you perform cooperative tasks in order to improve the relationships between colleagues?
  - Do you carry out activities that teach you strategies to study or learn better (how to make diagrams, summaries, etc.)?
  - When a problem occurs in class (not doing homework, bad behavior, fighting, …), do you dedicate the tutorial to solve it?
  - In general, are you satisfied with the tasks proposed by your tutor?
2. Emotional regulation questionnaire [18], adapted and validated into Spanish [23]. This scale is composed of 10 items (e.g. “1. I keep my emotions to myself”), which are rated along a Likert-type scale with 7 response options, where 1 indicates total disagreement and 7 indicates complete agreement. This questionnaire groups emotional regulation into two dimensions: Expressive suppression (items 1, 2, 3, and 4) and cognitive re-evaluation (items 5, 6, 7, 8, 9, and 10). Application of this instrument has shown acceptable internal consistency, although with a relatively low Cronbach’s alpha value of α = 0.633. Specifically, expressive suppression showed a value of α = 0.595, while cognitive re-evaluation obtained a value of α = 0.646.

### 2.3. Procedure

The research procedure employed in the present study attended to ethical principles for this type of research, set by Sales et al. [29] and Sikes et al. [30] for each of the stages described in the previous paragraph.

Thus, the study began with a prior explanatory phase in which the study centres were explained the purpose of the study by the research team, as well as the procedures to follow, and told that they would receive a report of the results. Once all access permissions were obtained from the students’ centres and families, interviewers who were appropriately trained to use the instrument explained its content to students, resolved doubts, and proceeded to its application for data collection. Preliminary verification of the quality of collected data was conducted before leaving the research setting, confirming that the associated items had been correctly completed and responses to the different items were valid.

The process of constructing the data matrix was also used as a means of verifying the quality of data collected in the questionnaires, excluding responses which demonstrated coding problems or a lack of clarity which impeded appropriate coding. Once data was analysed and a research report was developed which was adapted to the audience, a report explaining the results and educational recommendations was sent out to participating centres.

Finally, it is important to highlight that this study has respected the anonymity and confidentiality rights of the participants, assigning a code to each questionnaire applied. Likewise, the recommendations for research with humans established by the Declaration of Helsinki as well as the recommendations of the American Psychological Association have been considered. All these participants obtained the informed consent of their legal guardians.

### 2.4. Data Analysis

The statistical analysis software IBM SPSS v.23.0 was used to analyse the data. Frequencies and means were employed as the main statistics. The Student’s *t* test was used to carry out inferential analysis. This type of test was appropriate as the included sample fulfilled basic requirements for use of this statistical parametric (normality, homoscedasticity, and interdependence) with independent samples. It is an appropriate test for determining differences between the various variables analysed in the present study. Internal consistency of the instruments was determined through the Cronbach’s alpha coefficient. This is a classic index used in social science research. The reliability index was set at 95% for data analysis. Finally, it is important to note that all scales showed appropriate loadings in exploratory factor analysis and that significative correlations between the two dimension of emotion regulation were found.

## 3. Results

Table 2 shows the basic descriptions of the items related to the tutorial action that have been analyzed. Adequate values are obtained in the dispersion of the data in all the variables except in the group work (A = 3371; K = 9393), since it shows asymmetry and kurtosis values greater than 2. In this case, this variable is excluded in subsequent analysis.

Table 3 shows the basic descriptions of the items of emotion regulation. Adequate values are obtained in the dispersion of the data in all the items, since it shows asymmetry and kurtosis values lower than 2. Therefore, a normal distribution of the data is estimated.

Table 4 shows mean values for the dimensions of emotional regulation according to content worked on through tutorial action. Statistically significant differences were obtained for work with emotions (F(df) = 2.059, p < 0.05), with a greater mean value being observed for cognitive re-evaluation in students who worked on this aspect (M = 4.91, SD = 1.23) than for students who did not (M = 4.74, SD = 1.17). Significant differences were also observed for individuals working on socialisation (F(df) = −2.138, p < 0.05). This was reflected through identification of a lower mean in the dimension of expressive suppression for students who work on this type of content via tutorial action (M = 3.84, SD = 1.46), relative to students who had not worked on this aspect (M = 4.22, SD = 1.59). It is precisely through considering this variable that we see the greatest difference between means, this representing the importance of considering whether these aspects are worked on as an element of training or education. Finally, statistically significant differences were also revealed in students who tackled classroom coexistence problems through tutorial action (F(df) = −2.271, p < 0.05). These students reflected greater mean scores in relation to cognitive re-evaluation (M = 4.89, SD = 1.23) than students who had not worked on this type of aspect via tutorial action (M = 4.62, SD = 1.09), or had done so only occasionally through nonregulated and unplanned actions.

## 4. Discussion

First, it is a priority to discuss the percentages observed in the different items of the tutorial action in relation to the level of response of the students. It should be noted that approximately one in three schoolchildren conducts tutoring through one hour a week, an aspect that can negatively affect their academic and personal performance, as Calderón-Garrido et al. [2] show, although it is possible that this is being done in an infused way in the curriculum. On the other hand, it is observed that the activities most carried out in tutoring are those related to learning and study, which is associated with the traditional tutoring model established by Expósito [3], which is why no relationship with emotional regulation is subsequently observed. The second most worked content has been the issues related to socialization, since, as established by Talbot et al. [21], they are relevant to maintain a good climate in the classroom.

In line with the above, it should be noted that the content that showed the least frequency was that of emotions, which increases the value of beginning to work on emotional education in educational contexts—the tutorial action being a favourable space for it and that justifies the interest of this research work [8,10,15]. Lastly, it should be noted that there is a global satisfaction percentage with the actions carried out in high tutoring, since eight out of ten schoolchildren rated this item satisfactorily. However, this could be improved through the work of the least valued items, attending to a more holistic perspective of the personal, academic, and social development of the students [3,6].

Research on education and emotion reveals an ongoing relationship between emotions and the suitability or effectiveness of training processes. In this way, results obtained in the present study show that students who work on emotions in tutorials present higher means for cognitive re-evaluation. Obviously, it is important not to forget the fact that individual differences exist in relation to executive function, which enable these young people to deal with their negative emotions, as has been considered in the study conducted by Andrés et al. [31]. In other words, students who are engaged in interventions to improve their cognitive re-evaluation when faced with specific situations are capable of better interpreting those situations, with the outcome of modifying the emotional influence generated. This finding is congruent with and corroborates outcomes reported by previous studies, such as those by Garrosa et al. [32] and Andrés et al. [31], which denoted the importance of working on emotional education in classrooms. This aspect had already been indicated by Pena et al. [17] and has since been reiterated in numerous studies [33,34,35,36]. These cognitive re-evaluation processes do not only exert influence in the realm of emotions, they also facilitate the very regulation of cognitive processes and metacognition. This could easily be linked to the improvement of learning processes in general [37].

Further, such emotional work is considered to be efficient when approached through tutorial action [3,5] in order to obtain effective and appropriate learning results [6]. In this way, tutorial action becomes one of the settings in which emotional work is infused throughout all work tasks and topics. It serves to modify and resolve traditional challenges, directly integrating emotion within traditional areas of knowledge.

On the other hand, students who do not work on socialisation, which tends to be one of the most usual contents worked on in tutorial action, have higher scores for expressive suppression. This aspect has some very negative connotations for the personal and educational development of students [38], given that it implies the inhibition of emotional responses in specific situations. This inhibition is found at the root of all psychological and social problems related with poor emotional management [39]. Poor development from infancy could even lead to a sociopathic pathology.

Given that any problematic situation requires recognition and action to resolve it, the present results respond to a functional logic. Further, this solution is centred on the way in which individuals live situations and employ necessary strategies to autonomously resolve problems. Precisely, one of the issues that is most related with achieving successful outcomes from training processes is the capacity to improve the emotional maturity of the individuals involved in them [40].

Nevertheless, as shown by the results of the present study, work on all emotional education dimensions must not only be considered as an appropriate element in the development of individuals. It can also be considered as a preventative element of subsequent problems which could be associated with a lack of appropriate affective development. This is because individuals who are able to recognise their emotions, re-evaluate situations that raise emotional problems, and regulate these in order to respond appropriately, will be more successful in their learning and in all vital processes [25].

Another of the basic elements of educational development is related with sociability and coexistence. Joint responsibility between society and the educational system over the social education of students is continuously increasing [41]. Further, many potential problems specific to this context are related with the issues of a lack of coexistence or insufficient social competencies.

Social skills and their development are subject to training and educational processes, not only in a personal sense, but also a professional one. All training plans consider “being, knowing and knowing what to do” amongst their basic competencies. These considerations include aspects which refer to student development as people and, depending on their educational level, as professionals.

The sociability and social problems derived, such as a lack of coexistence, bullying, or a lack of social empathy, come from a lack of emotional education and poorly developed social skills. As a result, these aspects play a fundamental role in the development of educational processes. In this sense, as an outcome of the present study, it is shown that students who deal with coexistence problems through tutorial action present higher means for cognitive re-evaluation. This facilitates work on classroom conflict and helps students to learn how to better re-evaluate situations and generate appropriate emotional responses to them [1,25].

In other words, students who work on school coexistence problems during tutoring possess a greater capacity to consider issues in a “reasonable” way, re-evaluate them, and act accordingly. Through these processes, aspects are involved such as empathy, sociability, and everything associated with the “social being.” Thus, the re-evaluation of issues is typically approached by employing a different logic to that employed initially, with this new logic managing to include variables or considerations of a social type [2,24].

The present study did not identify significant differences in items such as dedication, group work, learning and study, or satisfaction. Such differences have been found in other similar studies [6], requiring further work in this line of research.

It can be generally concluded from the present study that, although it offers intuitive and informally expected results, it specifies and quantifies existing relationships between educational work on emotions as developmental elements of emotional regulation. This occurs through aspects such as cognitive re-evaluation and the capacity to offer emotionally appropriate responses when faced with problematic situations. In this way, the present study indicates the importance of working on emotions, not only as appropriate content for developing these competencies in students, but also as a preventative element of subsequent problems related with their capacity for social integration or to respond to problematic social issues [42]. Education and emotions, therefore, go hand in hand and represent a main responsibility of both training systems and the teachers who practice within them.

## 5. Conclusions

Answering the hypothesis raised in the study, it should be noted that this was partially fulfilled. Thus, it was shown that those students who carried out activities related to their emotions and the improvement of coexistence in tutoring had a greater cognitive reevaluation. Likewise, those schoolchildren who worked activities associated with socialization had less expressive suppression, which denotes better emotional regulation. Thus, the main conclusions of this study are below.

- On the one hand, students who work on emotions in tutorials have better cognitive re-evaluation. As a result of this, they will be more capable of interpreting issues to modify their emotional influence, or of employing the emotional realm to establish far more emotionally stable and satisfactory solutions.
- On the other hand, students who do not work on socialisation through tutorial actions present greater expressive suppression, inhibiting any emotional response, or not expressing certain responses. The present research derived the importance of working on all dimensions of emotional education as a preventative element of subsequent future problems.
- Further, students who work on coexistence through tutorial actions have better cognitive re-evaluation skills. This provokes emotional responses to different situations, in this way facilitating coexistence.

Finally, it must be highlighted that the present research offers important conclusions with respect to the emotional education and tutoring of students. This is because it establishes and details the relationship between tutoring work and student emotions as a developmental element of emotional regulation. It reveals that this relationship will be important for the cognitive re-evaluation of students and makes them capable of offering emotionally appropriate responses, when faced with diverse problematic issues or in anticipation of them. In other words, education that is based on emotions, whilst targeting emotions at the same time, becomes a preventative and palliative element of many problems found in educational settings. Thus, education and emotions go hand in hand, being a responsibility of both training systems and the teachers who practice within them.

## 6. Limitations

Finally, it is essential to indicate the main limitations presented by the developed study. Firstly, it is appropriate to indicate the study design. The present study was descriptive and cross-sectional in nature, which does not enable causal relationships to be established between studied variables—emotional regulation and tutorial action. However, it is also essential to point out that this design is effective for understanding the state of the issue, and it is useful for determining associations between variables, with the aim of setting the groundwork for future research in this area.

The instruments utilised should also be indicated as a central limitation. Acceptable Cronbach’s alpha coefficients were obtained in both cases, denoting good internal consistency. Nevertheless, values were not very high, which could detract to a certain extent from the validity of obtained data. As a result of this, there is a patent need to adapt the emotional regulation questionnaire to the school context, with the aim of facilitating understanding of the different items and obtaining better validity indices.

The third limitation resides in the study sample size and participant selection. The sample did not constitute a representative sample of the province of Granada given that it was selected by convenience. Despite this, it is worthwhile to indicate that the overall number of individuals interviewed corresponds to an acceptable number of participants for the number of items included in both questionnaires. Likewise, given that participants were selected according to natural groups at different educational centres, a certain degree of randomness of the sample could be concluded in accordance with that previously established by the scientific literature.

The variables themselves could be indicated as a limitation given that only two dimensions of emotional regulation were considered, alongside the factors mentioned which are linked to tutorial action. Thus, it would be interesting to consider as future perspectives other variables which could be linked to these elements. These could include emotional intelligence itself, school adjustment, academic performance, or family functioning.

On the other hand, the items associated with the tutorial action were assessed dichotomously, which may constitute a limitation for the study by restricting the response range. In addition to this, there may be a limitation in the students’ responses to the questions posed, both due to the social desirability of the answer and the presence of the tutor in completing the questionnaire. These aspects could be solved by adapting the scale associated with the tutorial action, being scored through a Likert-type scale, as well as extracting this information from another highly relevant group, such as teachers or those responsible for the educational centres.

## Figures and Tables

**Table 1:** Elements associated with Tutorial Action.

Dimension	Description
Dedication	Considering the weekly time spent in tasks associated with teacher-student tutoring
Emotions	A variable that is associated with the development of emotional intelligence, improving the understanding of emotions and their regulation
Group Work	Training tasks that allow students to acquire skills associated with working with peers
Socialization	Proposing dynamics that help develop social skills and build stronger relationships
Learning and Study	Variable associated with the work of learning strategies, such as the strategies of elaboration, organization, or regulation of effort
Coexistence	Actions linked to the improvement of the classroom climate and conflict resolution
Satisfaction	Assess the opinion of the students in relation to the actions carried out since a tutorial action, in order to adapt these to school demands

**Table 2:** Basic descriptive values of items related to tutorial action.

	Dedication	Emotions	Group Work	Socialisation	Learning and Study	Coexistence	Satisfaction
**M**	1.29	1.50	1.07	1.15	1.14	1.21	1.18
**SD**	0.45	0.50	0.25	0.35	0.34	0.41	0.38
**V**	0.21	0.25	0.06	0.12	0.12	0.16	0.14
**A**	0.86	0.01	3.37	1.95	1.85	1.39	1.67
**K**	−1.10	−1.99	9.39	1.82	1.96	−0.05	0.81

Note 1: A, Mean; SD, Standard Deviation; V, Variance; A, Asymmetry; K, Kurtosis.

**Table 3:** Basic descriptive values of items related to emotion regulation.

	I.1	I.2	I.3	I.4	I.5	I.6	I.7	I.8	I.9	I.10
**M**	4.09	3.33	3.65	4.43	4.32	4.87	5.43	4.58	4.58	4.99
**SD**	2.23	2.36	2.23	2.28	2.34	2.22	1.91	2.11	1.99	2.03
**V**	5.00	5.59	4.97	5.23	5.48	4.92	3.66	4.46	3.97	4.15
**A**	−0.06	0.42	0.22	−0.24	−0.26	−0.60	−1.09	−0.42	−0.40	−0.63
**K**	−1.40	−1.42	−1.33	−1.43	−1.46	−1.11	0.05	−1.09	−0.97	−0.83

Note 1: A, Mean; SD, Standard Deviation; V, Variance; A, Asymmetry; K, Kurtosis.

**Table 4:** Emotional regulation according to content worked on through tutorial action.

						*t* Test	
			N	Mean	SD	F	Sig.
**Dedication**	RE.SE	Yes	402	3.92	1.53	0.486	0.627
		No	167	3.85	1.42		
	RE.RC	Yes	402	4.83	1.22	0.085	0.933
		No	167	4.82	1.20		
**Emotions**	RE.SE	Yes	288	3.97	1.54	1.296	0.196
		No	284	3.81	1.43		
	E.RC	Yes	288	4.91	1.23	2.059	*
		No	284	4.74	1.17		
**Socialisation**	RE.SE	Yes	488	3.84	1.46	−2.138	*
		No	82	4.22	1.59		
	RE.RC	Yes	488	4.85	1.21	0.826	0.409
		No	82	4.73	1.21		
**Learning and study**	RE.SE	Yes	494	3.91	1.50	0.153	0.879
		No	80	3.88	1.48		
	RE.RC	Yes	494	4.83	1.21	0.030	0.976
		No	80	4.83	1.21		
**Co-existence**	RE.SE	Yes	448	3.87	1.50	−0.891	0.373
		No	125	4.00	1.44		
	RE.RC	Yes	448	4.89	1.23	2.271	*
		No	125	4.62	1.09		
**Satisfaction**	RE.SE	Yes	473	3.92	1.49	0.570	0.569
		No	101	3.82	1.51		
	RE.RC	Yes	473	4.85	1.23	0.790	0.430
		No	101	4.75	1.07		

Note 1: RE.SE. Emotional regulation – expressive suppression; RE.RC. Emotional regulation – cognitive re-evaluation; Note 2: *. Statistically significant difference (p < 0.05).
